# Metacognitive impairments extend perceptual decision making weaknesses in compulsivity

**DOI:** 10.1038/s41598-017-06116-z

**Published:** 2017-07-26

**Authors:** Tobias U. Hauser, Micah Allen, NSPN Consortium, Edward T. Bullmore, Ian Goodyer, Peter Fonagy, Peter Jones, Pasco Fearon, Gita Prabhu, Michael Moutoussis, Michelle St Clair, Kalia Cleridou, Hina Dadabhoy, Sian Granville, Elizabeth Harding, Alexandra Hopkins, Daniel Isaacs, Janchai King, Danae Kokorikou, Harriet Mills, Sara Pantaleone, Geraint Rees, Raymond J. Dolan

**Affiliations:** 1 Wellcome Trust Centre for Neuroimaging, University College London, London, WC1N 3BG United Kingdom; 2 Max Planck University College London Centre for Computational Psychiatry and Ageing Research, London, WC1B 5EH United Kingdom; 3 Institute of Cognitive Neuroscience, University College London, London, United Kingdom; 4 Department of Psychiatry, University of Cambridge, Cambridge, CB2 0SZ United Kingdom; 5 Cambridgeshire and Peterborough National Health Service Foundation Trust, Cambridge, CB21 5EF United Kingdom; 6 Medical Research Council/Wellcome Trust Behavioural and Clinical Neuroscience Institute, University of Cambridge, Cambridge, CB2 3EB United Kingdom; 7 ImmunoPsychiatry, GlaxoSmithKline Research and Development, Stevenage, SG1 2NY United Kingdom; 8 Research Department of Clinical, Educational and Health Psychology, University College London, London, WC1E 6BT United Kingdom; 9 Anna Freud National Centre for Children and Families, 12 Maresfield Gardens, London, NW3 5SU United Kingdom

## Abstract

Awareness of one’s own abilities is of paramount importance in adaptive decision making. Psychotherapeutic theories assume such metacognitive insight is impaired in compulsivity, though this is supported by scant empirical evidence. In this study, we investigate metacognitive abilities in compulsive participants using computational models, where these enable a segregation between metacognitive and perceptual decision making impairments. We examined twenty low-compulsive and twenty high-compulsive participants, recruited from a large population-based sample, and matched for other psychiatric and cognitive dimensions. Hierarchical computational modelling of the participants’ metacognitive abilities on a visual global motion detection paradigm revealed that high-compulsive participants had a reduced metacognitive ability. This impairment was accompanied by a perceptual decision making deficit whereby motion-related evidence was accumulated more slowly in high compulsive participants. Our study shows that the compulsivity spectrum is associated with a reduced ability to monitor one’s own performance, over and above any perceptual decision making difficulties.

## Introduction

Knowing what you did and how well you did it is crucial for achieving one’s goals and making adequate decisions^1^. Humans are burdened with imperfect perception and recollection, and this extends to the metacognitive ability to recognize such deficits. Despite this sub-optimality, we retain an ability to quantify the degree to which we can rely on our behaviour as represented by the feeling of confidence.

Confidence helps us determine how much credit we should assign to an information source, enabling us to calibrate our future behaviour. Metacognitive ability is thus important for good performance, and it is known that metacognitive training improves decision making^2^. However, there are considerable variations in metacognitive performance, i.e. how well humans are able to consciously judge their own performance^3–5^. Poor metacognitive skills, or insight, can have detrimental real-world consequences. For example, one might assign too much credit to a poorly informed decision or exhibit too little trust in a good decision. In extremis, impaired metacognition might lead to systematically bad decisions, for example continuously enacting the same behaviour regardless of outcome, as observed in obsessive checking^6^.

Obsessive-compulsive disorder (OCD) is a condition linked to metacognitive impairment. This disorder is characterized by intrusive thoughts and images (obsessions), and these are coupled to repetitive behaviours (compulsions) which serve to alleviate obsession-induced distress^7^. Initial theories of metacognitive impairments in OCD propose patients overestimate the credibility of their intrusions, believing their likelihood of becoming real^8,9^. Therapy for OCD often targets these (meta-) cognitive biases^6^. More recent accounts propose that metacognitive impairments are not restricted to intrusions, but also apply to memory recollection, although not unequivocally^10–15^. Thus, impairments in meta-memory are believed to drive repetitive checking, because low confidence in one’s own memory is likely to cause a repetition of a previously carried out action^16,17^. However, findings of lowered confidence in patients with OCD in cognitive domains other than memory^18–20^ suggest OCD patients might suffer from a more general impairment in metacognition.

Traditional studies of metacognition using questionnaires^11,14,21–25^ or subjective confidence ratings^10,12,13,15^ are subject to influences that may mimic a metacognitive impairment, such as systematic response biases in questionnaires and other confidence scales^26^. Here, we operationalize metacognition as the objective sensitivity of confidence ratings to discrimination performance, as defined by signal detection theory^27^. Metacognition thus reflects the degree of insight into one’s behaviour, i.e. how well one knows their own performance. This model-based measure is robust against general biases in rating behaviours (e.g. generally lower or higher ratings) and is independent of variability in perceptual decision making that can directly influence confidence ratings. The latter is of particular importance as OCD patients are reported to suffer from perceptual decision making difficulties^28,29^. By combining a computational model of metacognition together with experimentally controlled task difficulty, here we circumvent these limitations to single out contributing factors that selectively influence perceptual and metacognitive abilities^26^.

In this study, we probed metacognitive abilities along a recently proposed compulsivity spectrum^30,31^ using a perceptual decision making task in two groups of participants with either high or low obsessive- compulsive scores. These participants were carefully selected from a large cohort so as to match for potential confounding factors, such as depressive or anxiety symptoms^13^. The psychophysical detection task was continuously and automatically adapted for each participant to maintain constant performance levels, allowing us to study separate perceptual decision making and metacognitive differences. Using a hierarchical metacognition model, we analysed participants’ metacognitive efficiency, allowing us to map the objective sensitivity of a participant’s subjective beliefs (i.e., confidence) to actual underlying performance. Using this computational approach, we found that compulsivity is related to impairments in metacognitive efficiency, and that this was complemented by an independent perceptual decision making impairment.

## Methods

### Participants

We recruited forty participants from a large population-based sample of 2409 young people in London and Cambridge (U-CHANGE study; www.nspn.org.uk)^32,33^. We used a directed sampling approach, selecting twenty participants with high scores on an obsessive-compulsive measure (‘high compulsives’) and twenty participants with low obsessive-compulsive scores (‘low compulsives’). For this categorisation we used the PI-WSUR questionnaire^34^ (total score) as an index of compulsivity. The groups were selected so as to match in terms of age, gender, depression (using MFQ questionnaire^35^; relative symptom severity relative to population: low compulsivity: 31.5 ± 13.6 percentile, high compulsivity: 30.1 ± 16.2), and anxiety levels (using RCMAS questionnaire^36^; relative symptom severity: low compulsivity: 31.6 ± 12.1 percentiles, high compulsivity: 28.7 ± 14.5). The groups also did not differ in IQ (using vocabulary and matrix subtests of WASI battery)^37^ and impulsivity (BIS questionnaire)^38^. Participants that reported any psychiatric or neurological disorders were excluded a priori. All participant had normal or corrected-to-normal vision.

The selected groups differed strongly in their compulsivity scores, but were otherwise well matched across all other psychiatric dimensions (Table 1). Two high compulsive participants were excluded from data analysis due to difficulties with the task (staircase failed to converge). The study was approved by the UCL research ethics committee (No. 6218/001) in accordance with the Declaration of Helsinki and all participants gave written informed consent.

**Table 1:** Participants with high and low compulsivity scores.

	Low compulsives	High compulsives	
age	21.40 ± 2.52	20.75 ± 2.34	t (38) = 0.85, p = 0.403
gender (f/m)	13/7	14/6	χ (1) = 0.114, p = 0.736
IQ (WASI total)	115.60 ± 10.91	115.40 ± 9.80	t (38) = 0.06, p = 0.952
PI-WSUR*	5.25 ± 4.00	50.18 ± 18.28	t(38) = 10.74, p < 0.001
MFQ*	19.12 ± 8.94	19.36 ± 11.67	t (38) = 0.07, p = 0.942
RCMAS*	20.70 ± 10.09	18.70 ± 10.65	t (38) = −0.61, p = 0.545
BIS	58.30 ± 6.87	59.04 ± 9.74	t (38) = −0.28, p = 0.782

Two groups of participants were recruited from a population-based database, based on their compulsivity scores (PI-WSUR). The groups were matched for other psychiatric dimensions, especially depression (MFQ) and anxiety (RCMAS). Groups did not differ in age, gender, IQ, or impulsivity (BIS). (mean ± SD).

*Data used for recruiting.

### Task

**Figure 1:**
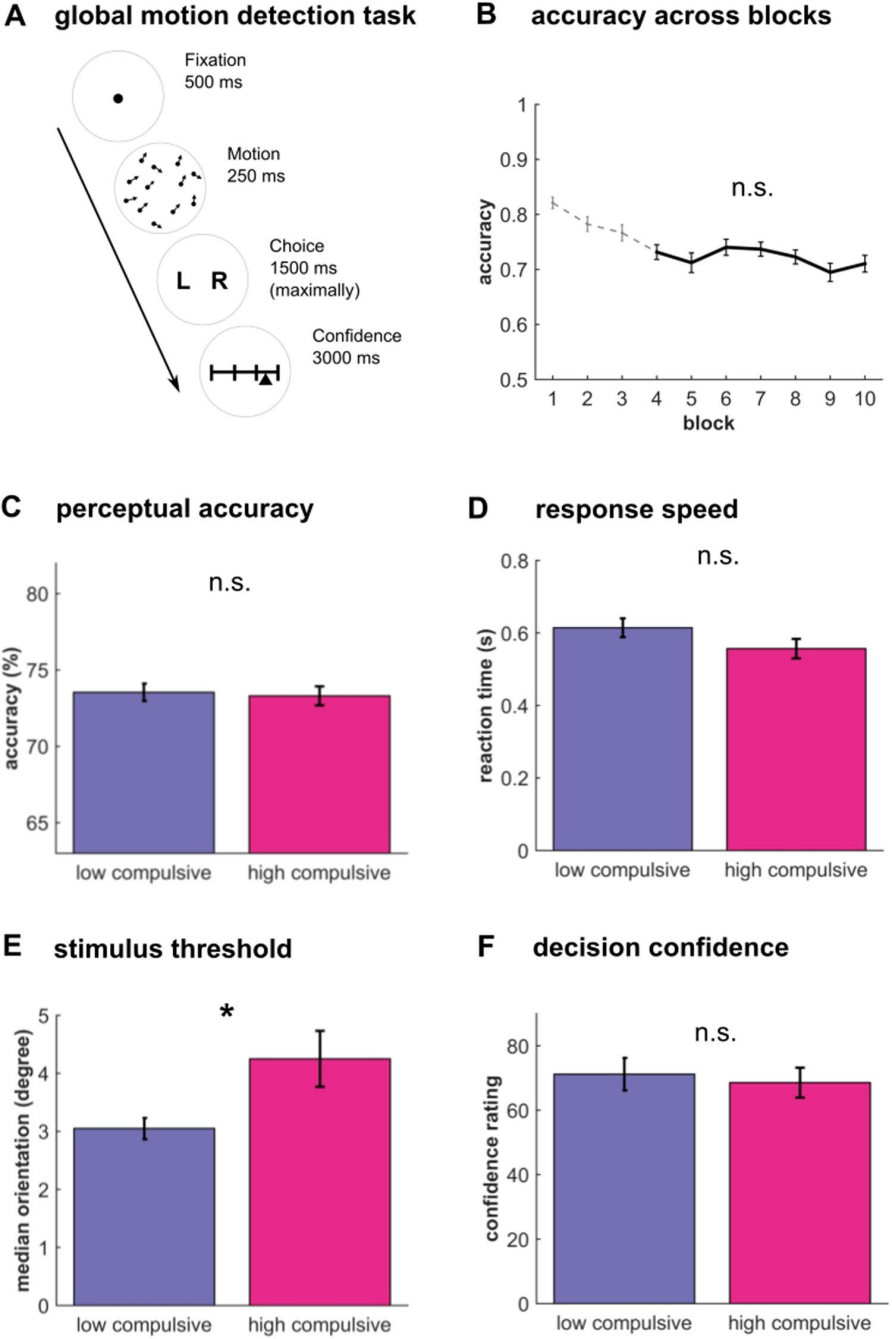
Metacognition task performance. High and low compulsive participants performed a metacognition task. (**A**) Participants saw a cloud of dots moving with a defined mean motion orientation plus added random movement noise. After participants’ categorical judgement of the main direction of stimuli they then had to rate their confidence using a visual slider. (**B**) A staircase procedure ensured that performance was stable (the first three block were omitted (dotted line), because stability was not yet reached). This staircase ensured that both groups performed at the same level (**C**) and did not differ in their mean reaction times (**D**). Mean confidence ratings were similar between groups (**F**), but the sensory signal was significantly stronger in high compulsives (**E**), indicating a poorer perceptual decision making performance in high compulsive participants. Bar plots: mean ± 1s.e.m; *p < 0.05; n.s. p > 0.05.

We used a metacognition task based on a global motion detection paradigm, similar to that reported previously^3,39,40^. The task (Fig. 1A) consisted of 140 trials subdivided into 10 blocks, with short breaks between blocks. On each trial, participants judged whether the global motion of the randomly moving dots was directed left- or rightwards relative to vertex. Subsequently, participants had to indicate their confidence using a visual analogue scale, where 0 indicated a guess and 100 total certainty. To prevent motor preparation, the starting point of the confidence slider was randomly adjusted to +/−12% of the scale midpoint. Before the main task, participants completed a short training and were also instructed to use the entire confidence scale for their confidence ratings. The task was implemented using Psychtoolbox 3 (www.psychtoolbox.org) in MATLAB (MathWorks Inc.).

The motion signal consisted 1100 black dots presented for 250 ms. The motion direction of the dots was determined using a mean motion angle (‘orientation’, in degrees from vertical movement) plus Gaussian noise with a standard deviation of 15 degrees. The mean motion orientation of the stimulus was adjusted on each trial so that participants performed consistently around 71% in an adaptive 2-up-1-down staircase procedure^41^. This ensured that detection performance (*d*’) of all participants was roughly equal enabling a higher sensitivity for assessing metacognitive performance^26,42^. A full description of the motion stimuli and the staircase procedure can be found in Allen *et al*.^3^.

### Behavioural analysis

To assess performance of our groups we compared confidence ratings, accuracy, signal strength (stimulus motion orientation) and reaction times using independent-sample t-tests. To allow staircase stabilization, we discarded the first 30% of trials (three blocks total). Additionally, any missed trials were excluded from all analyses. Repeated-measures ANOVA confirmed that performance was stable (Fig. 1B) for the remaining seven blocks (F (6, 222) = 1.15, p = 0.337).

### Metacognition model

Metacognition reflects an ability to consciously access one’s own performance, which in this context refers to explicitly distinguishing between a correct and an incorrect response. Traditional approaches for analysing metacognition (e.g., mean confidence rating or correlating confidence and accuracy) are subject to bias by perceptual performance such that it is impossible to tease apart metacognitive from perceptual difficulties or response biases^26^. Recently developed model-based approaches that control for these confounds circumvent these difficulties and provide an unbiased estimate of metacognition^26^.

Here, we use the metacognitive efficiency (*M-ratio*), an established marker of metacognition that is based on signal detection theory^42^. The *M-ratio* is calculated as the ratio between the second order, or type-II metacognitive sensitivity *meta-d’* and the perceptual sensitivity *d*’^42^. *M-ratio* controls accurately for potential perceptual differences as well as response biases, and is thus superior to model-free approaches. This is particularly critical in compulsivity as OCD patients have previously been found to have worse perceptual decision making performance^28^.

To estimate the *M-ratio*, we used a recently described hierarchical modelling approach that is implemented in a freely available toolbox (HMeta-d toolbox^43^, https://github.com/smfleming/HMM). This toolbox is a Bayesian extension of the widely used metacognitive efficiency model^39,44–48^ by Maniscalco & Lau^42^, and allows estimation and comparison of group-level parameters. This is particularly critical for studies with vulnerable groups, such as ours, because hierarchical models have regularising effects on the parameter estimates and allow adequate parameter estimation within relatively few trials, as in our study. Moreover, the Bayesian nature of this model naturally provides information about parameter uncertainty, which can then be used for group comparisons^43^. The model is built so that it estimates a group-level metacognitive efficiency (*M-ratio* = *meta-d'*/*d'*), which in turn governs individual participants’ behaviour. The optimisation of the (log-transformed) metacognitive efficiency, rather than the *meta-d’*, was used in our study because the latter is influenced by *d’* which could bias metacognition results if *d’* were different^43^. Because the model renders both *d’* and *meta-d’* in standard signal detection units, their ratio describes how much of the available signal (i.e., their perceptual sensitivity) is captured by confidence ratings, capturing this potential bias^42^. Simulation studies have demonstrated that this model provides more adequate parameter estimates than previous models, especially in situations involving relatively few trials, such as ours^43^.

The parameters were estimated using Markov-Chain Monte-Carlo methods (MCMC, here: 3 chains of 10’000 samples each, burn-in of 1000 samples) as implemented in JAGS (http://mcmc-jags.sourceforge.net). MCMC sampling methods are reliable methods for parameter estimation robust to local minima and parameter recovery studies show a reliable parameter estimation for a given model^43^. We used the wide standard priors for the model that have been found to be sensitive to detect group differences^43^. Model convergence was ensured by inspecting MCMC chains as well as checking that the $\hat{R}$ convergence measures for all parameters were <1.1.

We followed the standard group comparison approach by estimating each group separately and then compared the posterior group distributions in metacognitive efficiency^43^. To assess significance we computed the difference of the group posteriors and compared the overlap with 0 of the resulting distribution (similar to a classical or frequentist statistical test, it assesses the probability of the difference between the groups to be 0), as well as the 95% high density intervals of the difference distribution (analogous to confidence intervals).

### Perceptual decision making model

Besides our comparison of a metacognitive ability between low and high compulsive participants, we were interested in whether we would replicate an independent, perceptual decision making deficit. Such an impairment was previously reported^28,29^, and our finding of an increased stimulus strength in high compulsives (see results) pointed towards a similar impairment.

Drift-diffusion models (DDM) are widely used to investigate perceptual decision making, can successfully capture underlying neural decision processes^49^, and generalise beyond perceptual decision making^50–52^. In keeping with the previous study on perceptual impairments in OCD^28,53^, we used an hierarchical version of a drift diffusion model^54^. The hierarchical drift diffusion model (HDDM)^55^ estimates group model parameters using MCMC, similar to the metacognition model described above, and thus provides robust parameter estimates.

We compared drift diffusion models with different parameterisations in order to determine the best-fitting model, which was then used for group comparisons. As per standard settings in the HDDM toolbox, all models were specified with the following free parameters: a drift rate *v* determines how rapidly evidence accumulates over time, the decision threshold *a* indicates the information threshold needed to commit to a decision, and the non-decision time *t* captures the decision-independent processing time. Critically, because we controlled for performance by adjusting signal strength (stimulus motion orientation) in our task, we used a regression analysis that allows *v* to be modulated by signal strength at every trial. This approach was used because is well known that stimulus strength directly influences an accumulation of evidence^49^.

To assess group differences, we entered both groups into the same hierarchical model, but used group membership to predict differences in the model parameters (implemented in the regression). This deviates from the metacognitive analysis, in which we estimated both groups separately. We applied this approach, because the HDDM^55^ offers the possibility to explicitly model a group factor, a feature not yet implemented in the metacognition toolbox^43^. Such an approach can help to further increase the robustness of the parameter estimates^55^.

We assessed different models where (i) group influenced drift rate directly, (ii) group and orientation effect on drift rate interacted (i.e. group predicts how strong orientation affects drift rate), (iii) group has a separate effect on decision threshold *a*; and (iv) group interacted with orientation effect on drift rate as well as a separate group effect on threshold. Models were compared using deviance information criterion (DIC)^55^, and posterior group parameters of the best-fitting model were further assessed.

We found that the best-fitting model was characterised by a regression model that incorporated both, influences of stimulus strength as well as group and their interaction:

$$v\sim orientation+group+orientatio{n}^{\ast }group,$$

where *orientation* depicts stimulus strength, *group* describes whether a subject belongs to the high or low group, and * depicts their interaction. The group level parameters of this winning model were *v*_*intercept*_ = 0.03 ± 0.26 (group mean ± group standard deviation), *v*_*orientation*_ = 0.27, *v*_*group*_ = 0.22, *v*_orientation*group_ = −0.13, *a* = 1.37 ± 0.14, *t* = 0.19 ± 0.11, and the DICs of all models are depicted in Table S1.

## Results

### Behavioural performance

**Figure 2:**
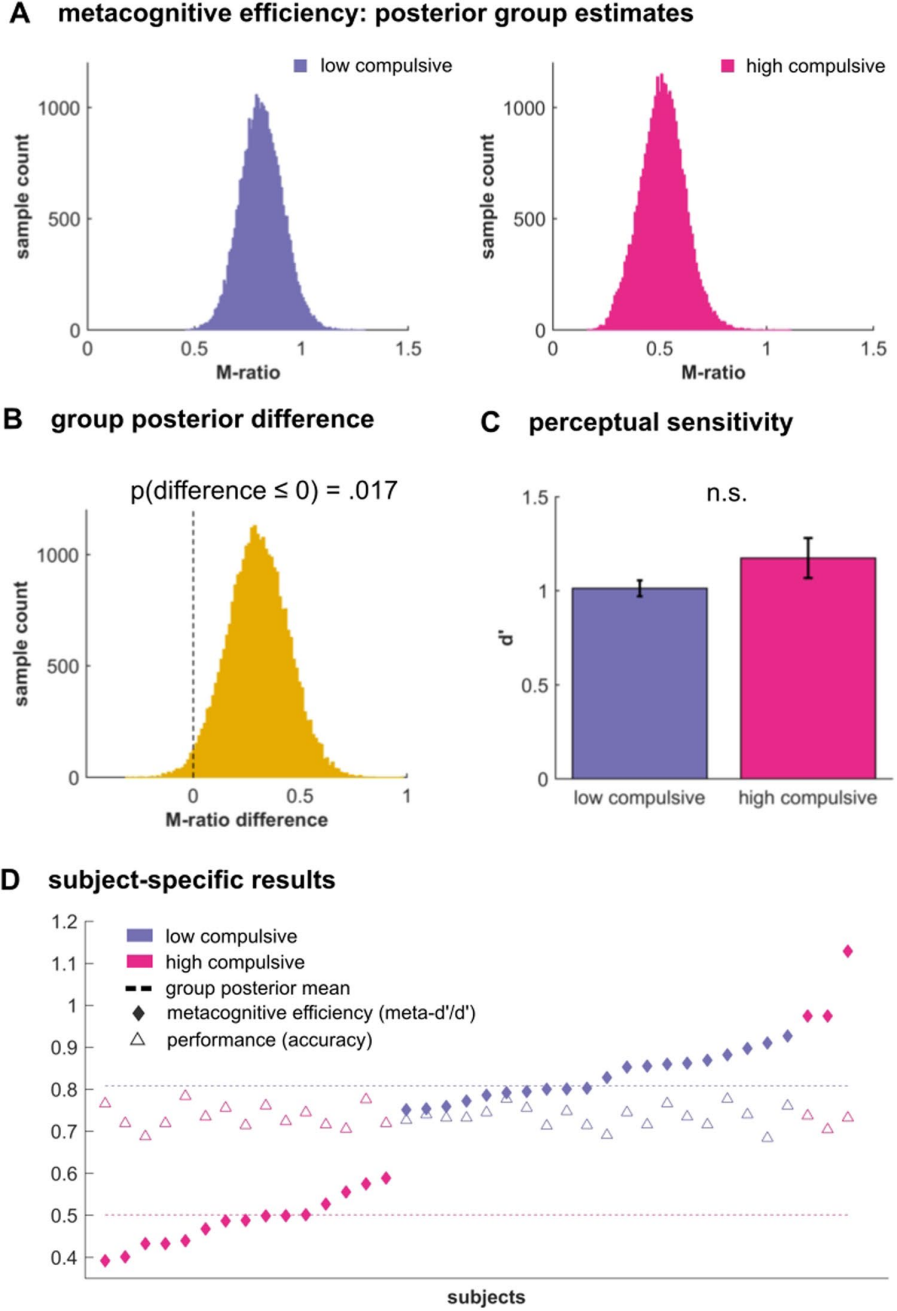
Metacognitive impairments in high compulsives. (**A**) Group posterior of metacognitive efficiency (*M-ratio*) for high and low compulsive participants revealed that high compulsive participants are significantly worse in their metacognitive abilities (**B**). This is not due to perceptual differences, because we controlled for performance, also indicated by the absence of a difference in the perceptual performance (*d’*, **C**). (**D**) An illustration of the individual metacognitive efficiencies (diamonds) reveals that all but three participants from the high compulsive group perform worse than the low compulsives. There were no systematic biases in the accuracy (triangles) across the groups which highlights that metacognitive biases are not driven by perceptual difficulty. However, it must be noted that the metacognitive efficiencies depicted here are derived from a hierarchical model, and can thus not easily be interpreted or compared individually (i.e., they are not statistically independent). Bar plots: mean ± s.e.m.; n.s. p > 0.10.

To attain a stable proportion of correct and incorrect responses for all participants we adapted the difficulty of the dot motion paradigm (Fig. 1A) by adjusting the motion orientation of the stimuli using a staircase procedure. The groups thus did not differ in response accuracy (Figs 1C and 2D; low compulsives: 73.54 ± 2.54; high compulsives: 73.31 ± 2.64; t(36) = 0.28, p = 0.780). Additionally, they did not differ in response latencies (Fig. 1D; low compulsives: 0.61 s ± 0.12; high compulsives: 0.56 s ± 0.11; t(36) = 1.55, p = 0.131). However, the stimulus motion orientation (median signal across trials), was significantly greater in high compared to low compulsive participants (Fig. 1E; low compulsives: 3.05 degrees ± 0.83; high compulsives: 4.25 ± 2.05; t(36) = −2.42, p = 0.021). This means that high compulsive participants required a stronger motion orientation signal to perform at the same error rate as the low compulsives, indicating a weaker perceptual detection performance.

Comparing mean confidence rating we found no significant difference (Fig. 1F; low compulsives: 71.17 ± 22.51; high compulsives: 68.56 ± 19.64; t(36) = 0.38, p = 0.706). This means that high compulsive participants were neither more, or less, biased in reporting subjective confidence. Mean confidence, however, gives little insight into how well participants can consciously monitor their performance. To examine metacognitive differences between the groups, we thus used a hierarchical metacognition model.

### Metacognitive impairments in high compulsive participants

We used a hierarchical metacognition model^43^ to assess group metacognitive efficiency (*M-ratio*). This signal detection theoretic measure captures the degree to which participants exploit a perceptual signal for their confidence judgement by controlling for potential confounds, such as performance or rating biases^42^. Metacognitive efficiency equals 1 if an agent has full access to their perceptual performance, whereas values below 1 mean that confidence reports are suboptimal and cannot access full perceptual information. The hierarchical nature of this model allows robust estimates of group level metacognitive efficiency and also allows comparison of these efficiencies between groups^43^.

Our computational modelling revealed that low compulsive participants have a mean metacognitive efficiency (*M-ratio*) of 0.814 (Fig. 2A, left panel), whereas high compulsive participants have a ratio of 0.512 (Fig. 2A, right panel). This means that low compulsive participants exploit about 80% of the perceptual signal for their metacognitive judgement. High compulsive participants, however, only use approximately 50% of the perceptual signal for their metacognitive judgement. Interestingly, depicting the individual estimates reveals that all but three participants from the high compulsive group performed worse than low compulsives (Fig. 2D).

A comparison of group posteriors revealed that the metacognitive efficiency was significantly lower in high compulsive participants (Fig. 2B; p(difference ≤0) = 0.017; equivalent to a one-sided significance test; 95% confidence intervals = 0.031–1.000). Importantly, this was not due to an impaired perceptual performance, as there was no significant group difference in their *d*’ (Fig. 2C; t (36) = 1.46, p = 0.153). A qualitatively similar result was obtained when approximating metacognitive sensitivity using a behavioural measure, which however is not robust to the aforementioned biases (Figure S1). These findings suggest that high compulsive participants have worse conscious access to their performance over and above any perceptual decision making impairments or response biases.

### Lower drift rate in high compulsives impairs perceptual decision making

Our finding of an increased motion signal in high compulsive participants suggests that these participants also have a perceptual decision making difficulty. To understand the processes underlying this impairment and to extend previous studies that found similar difficulties in OCD patients^28,29^, we used a hierarchical drift diffusion model^55^. Model comparison (Table S1) revealed that the drift rate was modulated by task difficulty, as reflected in stimulus motion orientation. A model with a group factor (low, high compulsives) that modulates drift rate and its interaction with stimulus orientation, but not decision threshold, performed best.

**Figure 3:**
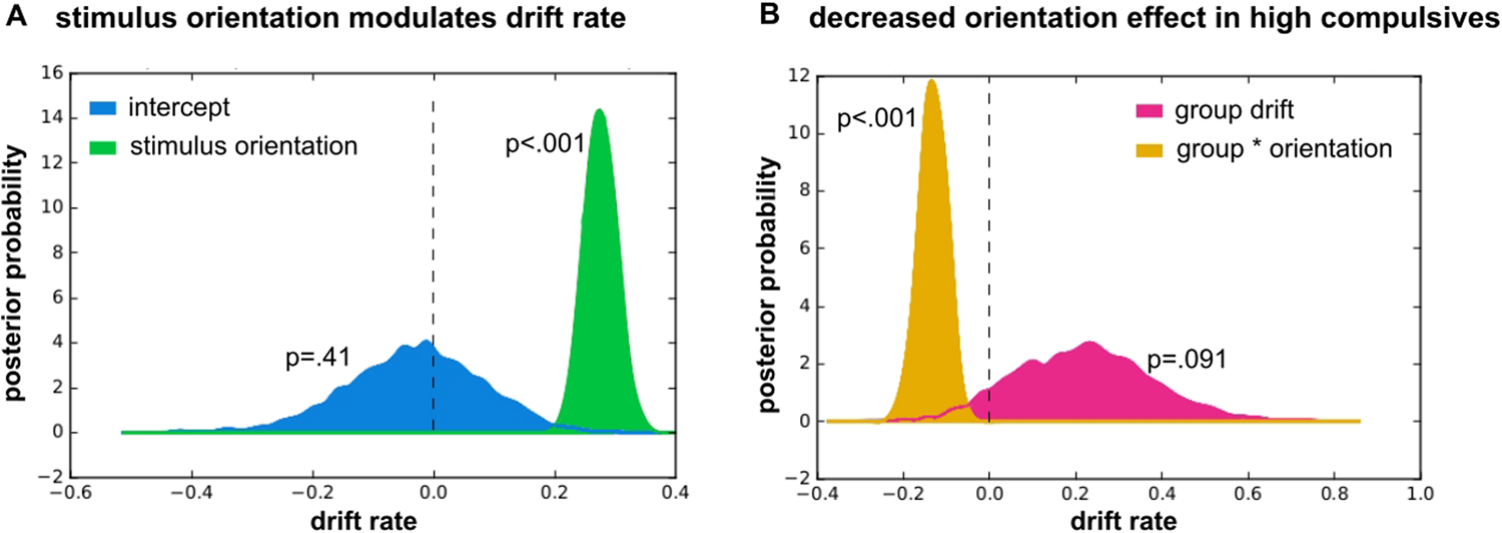
Stimulus processing is altered in high compulsive participants. (**A**) Signal strength (stimulus motion orientation) significantly increases drift rate across both groups (green). This effect entirely accounts for drift rate, as the orientation-independent drift rate (‘intercept’, blue) is not significantly different from 0. (**B**) The groups differ in in how much the stimulus motion orientation affects the drift rate: high compulsive participants benefit significantly less from an increasing stimulus orientation (orange). There is no additional effect of group on the drift rate (pink).

To understand more precisely how the groups differ in their perceptual decision making, we evaluated the posterior model parameters of the best-fitting model. A highly significant influence of orientation on drift rate (Fig. 3A; p(*v*_*orientation*_ ≤ 0) <0.001) confirmed that stimulus difficulty directly influences evidence accumulation. The group factor had a highly significant impact on the relationship of stimulus orientation to drift rate (Fig. 3B; p(*v*_orientation*group_ ≥ 0) <0.001), meaning that high compulsive participants benefited less from the stimulus strength. The absence of a main effect of group on the drift rate suggests that there are no additional group-factors impacting the drift rate (Fig. 3B; p(v_group_ ≤ 0) = 0.091).

## Discussion

A longstanding tradition associates compulsivity with impairments in metacognition, but until now such metacognitive deficits have not been formally examined using a computational approach. In this study, we provide the first evidence that compulsivity is linked to an impairment in metacognitive abilities, independent of an additional impairment in perceptual decision making. This suggests that people with high compulsive traits are worse at introspectively monitoring their perceptual performance, and suffer from a degraded impact of sensory evidence on confidence.

Metacognition has traditionally been characterised as “thinking about thinking” or insight, a form of conscious monitoring or introspection about one’s decisions and experiences^1^. Humans differ considerably in their metacognitive abilities^3,4^. Metacognitive investigations in OCD have mainly focused on biases in stimulus-outcome beliefs, for example that which an intrusive thought is likely to instantiate^9^. Later accounts focused on memory-related confidence judgements, although with mixed results and heterogeneous approaches^10–15^, suggesting that OCD patients are not impaired in their memory, but in their confidence about their memory. Here, we expand on this research by showing that compulsive participants’ impairments are not restricted to biased beliefs or lowered confidence. Instead, we show that for high compulsive participants metacognitive judgements are less efficient, i.e. they are generally worse at accessing their own performance, a finding that holds when controlling for general response biases or perceptual decision making difficulty. This is of importance because it shows that compulsivity is related to impairments in metacognition, which sheds new light on the previous findings and theories. An impaired conscious access to one’s own performance can directly deteriorate the attitude towards intrusive thoughts and memories, as a poor monitoring system might induce a general distrust into one’s perceptions and recollections, and thus fosters distrust in memory recollection and an engagement in compulsive safety behaviours. Moreover, the recent finding that noradrenaline modulates metacognition suggests novel interventions to improve metacognitive abilities in compulsive patients^40^.

As reported in OCD patients^28,29^, we found that high compulsive participants also exhibit perceptual decision making impairments in the visual domain. This was expressed in our task as an increased stimulus motion orientation (i.e. signal strength), and our computational modelling related this impairment to a lower accumulation of sensory evidence, in accord with this previous study^28^. It is interesting to speculate how this perceptual decision making difficulty might be related to the metacognitive impairments observed here. In the simplest case, these impairments could be completely independent of one another, so that compulsivity is contributed to by a lower metacognitive efficiency as well as a lower perceptual decision making sensitivity. Alternatively, perceptual decision making impairments could indirectly affect metacognition in a bottom-up manner by also influencing a post-decision evidence accumulation process^39,56–58^. However, it is unclear how the increased signal strength for high compulsives would influence a post-decision accumulation. Lastly, a perceptual decision making difficulty could be a top-down consequence of impaired metacognition, where impaired metacognition alters the amount of evidence a participant needs to make a decision. This in turn could impact their behaviour in perceptual decision making tasks, such that they only decide once they have consciously perceived enough information, leading to an increased need for greater signal strength.

We focused on ‘healthy’ participants, selected from a large population-based sample, who scored high or low on a compulsivity scale. This had the advantage of controlling for psychiatric dimensions that are often comorbid with compulsivity, such as depression and anxiety. This is important given that metacognitive impairments are suggested to be symptomatic for many psychiatric disorders^59^. Thus, our experimental strategy allows us to be confident that observed differences are solely driven by compulsivity, but not by other psychiatric traits. Additionally, our replication of a perceptual decision making impairment similar to the one found in patients with OCD^28^ speaks to a conceptualisation of compulsivity in terms of as a spectrum, rather than as a categorical entity^30,31^. However, future studies of patients with OCD will be necessary to ascertain whether similar processes are impaired in participants with clinically relevant compulsivity.

In summary, we show that a compulsivity spectrum identified in the general population is linked to impairments in metacognitive efficiency. This impairment is expressed over and above an effect due to perceptual decision making difficulty. Our findings provide the first computational evidence that metacognition is impaired in compulsivity and thus clarify the relationship between compulsivity, perceptual performance and conscious insight.

### Data availability

The datasets analysed during the current study are not publicly available because it is not foreseen in ethics permission, but are available from the corresponding author on reasonable request.

## Electronic supplementary material

Supplementary Information
