# Publisher Correction: Metacognitive impairments extend perceptual decision making weaknesses in compulsivity

**DOI:** 10.1038/s41598-018-23433-z

**Published:** 2018-04-11

**Authors:** Tobias U. Hauser, Micah Allen, NSPN Consortium, Edward T. Bullmore, Ian Goodyer, Peter Fonagy, Peter Jones, Pasco Fearon, Gita Prabhu, Michael Moutoussis, Michelle St Clair, Kalia Cleridou, Hina Dadabhoy, Sian Granville, Elizabeth Harding, Alexandra Hopkins, Daniel Isaacs, Janchai King, Danae Kokorikou, Harriet Mills, Sara Pantaleone, Geraint Rees, Raymond J. Dolan

**Affiliations:** 1 Wellcome Trust Centre for Neuroimaging, University College London, London, WC1N 3BG United Kingdom; 2 Max Planck University College London Centre for Computational Psychiatry and Ageing Research, London, WC1B 5EH United Kingdom; 3 Institute of Cognitive Neuroscience, University College London, London, United Kingdom; 4 Department of Psychiatry, University of Cambridge, Cambridge, CB2 0SZ United Kingdom; 5 Cambridgeshire and Peterborough National Health Service Foundation Trust, Cambridge, CB21 5EF United Kingdom; 6 Medical Research Council/Wellcome Trust Behavioural and Clinical Neuroscience Institute, University of Cambridge, Cambridge, CB2 3EB United Kingdom; 7 ImmunoPsychiatry, GlaxoSmithKline Research and Development, Stevenage, SG1 2NY United Kingdom; 8 Research Department of Clinical, Educational and Health Psychology, University College London, London, WC1E 6BT United Kingdom; 9 Anna Freud National Centre for Children and Families, 12 Maresfield Gardens, London, NW3 5SU United Kingdom

Correction to: *Scientific Reports* 10.1038/s41598-017-06116-z, published online 26 July 2017

The original version of this Article contained an error in the order of the author names, which were incorrectly given as ‘Tobias U. Hauser, Micah Allen, Geraint Rees, Raymond J. Dolan & NSPN Consortium’.

This error has now been corrected in the HTML and PDF versions of this Article.

