# Supplementary material for: Metacognitive impairments extend perceptual decision making weaknesses in compulsivity
Source: Sci Rep. 2017 Jul 26;7:6614. doi: 10.1038/s41598-017-06116-z (PMC5529539; doi:10.1038/s41598-017-06116-z)
Supplement: Supplementary file 1 — Supplementary Information [file 41598_2017_6116_MOESM1_ESM.pdf]

Metacognitive impairments extend perceptual decision making weaknesses in compulsivity

Tobias U. Hauser, Micah Allen, NSPN Consortium, Geraint Rees & Raymond J. Dolan

Supplementary Information

Content:

Table S1. HDDM model comparison

Figure S1. Binned confidence ratings

Neuroscience in Psychiatry Network (NSPN) Consortium author list

Table S1. HDDM model comparison. Model comparison revealed an improved fit for models that allow the orientation of the stimuli to modulate drift rate (‘orientation→drift’). Additionally, models that included the group membership improved model fit. This was especially so when the group factor interacted with the orientation effect on the drift rate (orientation*group→drift). Best fitting model highlighted in bold.

**model**	**DIC**
basic model	3670
orientation→drift	3524
orientation+group→drift	3523
**orientation*group→drift**	**3517**
orientation→drift, group→threshold	3524
orientation*group→drift, group→threshold	3519

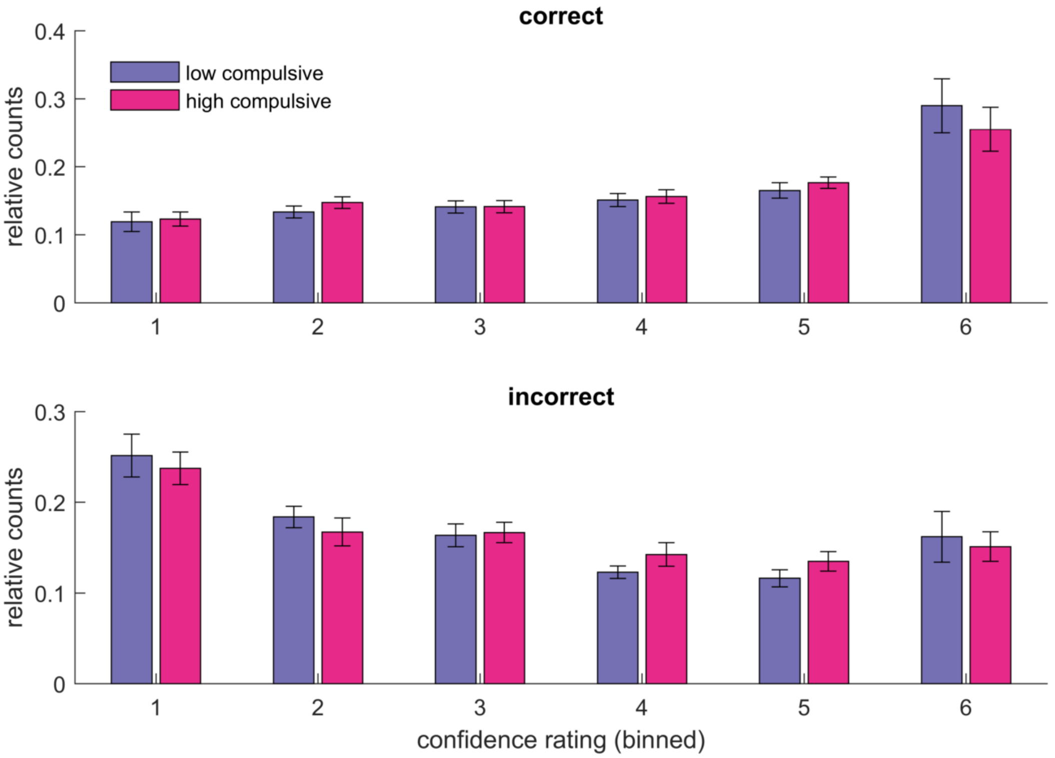

Figure S1. Binned confidence ratings. Deriving metacognitive measures without using a computational model is difficult, because these confidence ratings are inherently subject to a range of distortions, including rating and perceptual performance biases. However, to approximate the model-based results that we found in our main analysis, we used linear regressions to predict the relationship between perceptual performance (correct vs incorrect) and binned confidence rating (left: lowest confidence, right: highest confidence). We used a summary statistics approach to estimate the slope of how often a particular confidence was expressed, given true performance. These slopes were independently estimated for correct and incorrect trials, and the difference of these slopes were then compared between the groups. Such a measure is a rough indicator for how much a participant distinguishes between correct and incorrect decision and can only be used as a model-free approximation for metacognitive performance. Indeed, we found a positive slope for correct decisions (low compulsives: $z(196)=3.40$, $p<.001$; high compulsives: $z(167)=3.55$, $p<.001$) and a negative slope for incorrect decisions (low compulsives: $z(41)=-2.39$, $p=.017$; high compulsives: $z(24)=-2.68$, $p=.007$) for both groups. A group comparison showed that high compulsive participants show (at a trend level significance) less distinction in the slopes between correct and incorrect trials in terms of confidence ratings (slope difference: low compulsives: 0.05 ± 0.02; high compulsives: 0.04 ± 0.03; $z(456)=1.91$, $p=0.056$). This finding supports the main analysis of a lower metacognitive efficiency in high compulsive participants.

Neuroscience in Psychiatry Network (NSPN) Consortium author list

Principal investigators:
Edward T. Bullmore [5-8] (chief investigator from 01/01/2017)
Ian Goodyer [5,6] (chief investigator until 01/01/2017)
Peter Fonagy [9]
Peter Jones [5,6]

Associated faculty:
Pasco Fearon [9]

Project managers:
Gita Prabhu [1,2]

Postdoctoral research associates and associated research fellows:
Michael Moutoussis [1,2]
Michelle St Clair [5]

Research assistants:
Kalia Cleridou [10]
Hina Dadabhoy [10]
Sian Granville [10]
Elizabeth Harding [10]
Alexandra Hopkins [1,2,10]
Daniel Isaacs [10]
Janchai King [10]
Danae Kokorikou [10]
Harriet Mills [10]
Sara Pantaleone [10]

[5] Department of Psychiatry, University of Cambridge, Cambridge CB2 0SZ, United Kingdom
[6] Cambridgeshire and Peterborough National Health Service Foundation Trust, Cambridge, CB21 5EF, United Kingdom
[7] Medical Research Council/Wellcome Trust Behavioural and Clinical Neuroscience Institute, University of Cambridge, Cambridge CB2 3EB, United Kingdom
[8] ImmunoPsychiatry, GlaxoSmithKline Research and Development, Stevenage SG1 2NY, United Kingdom
[9] Research Department of Clinical, Educational and Health Psychology, University College London, London WC1E 6BT, United Kingdom
[10] Anna Freud National Centre for Children and Families, 12 Maresfield Gardens, London NW3 5SU, United Kingdom

Conflict of interest for NSPN members:

E.T.B. is employed half-time by the University of Cambridge and half-time by GlaxoSmithKline and holds stock in GlaxoSmithKline.
